# Scleral buckling with adjuvant pneumatic retinopexy versus scleral buckling alone for rhegmatogenous retinal detachment

**DOI:** 10.1038/s41598-024-55999-2

**Published:** 2024-03-04

**Authors:** Young Hoon Jung, Kyu Hyung Park, Se Joon Woo, Kwangsic Joo, Min Seok Kim

**Affiliations:** 1 Department of Ophthalmology, Seoul National University College of Medicine, Seoul National University Bundang Hospital, 173-82 Gumi-ro, Bundang-gu, Seongnam-si, Gyeonggi-do 13620 South Korea; 2 Department of Ophthalmology, Seoul National University Hospital, Seoul, South Korea; 3 Department of Ophthalmology, Seoul National University College of Medicine, Seoul, South Korea

**Keywords:** Retinal diseases, Vision disorders

## Abstract

To compare the efficacy of scleral buckling with adjuvant pneumatic retinopexy (SB with PR) and scleral buckling (SB) alone for primary rhegmatogenous retinal detachment (RRD). This retrospective and comparative study included patients who underwent SB with PR (n = 88) or SB alone (n = 161) for primary RRD. The primary anatomical success rate for SB with PR was 81.8%, whereas that for SB alone was 80.7% (*P* = 0.836). Among patients who achieved primary anatomical success, those in the SB with PR group showed postoperative epiretinal membrane (ERM) formation more frequently than those in the SB alone group (11 of 72 [15.3%] vs. 6 of 130 [4.6%]) (*P* = 0.009). The mean time to subretinal fluid absorption was not significantly different between the SB with PR and SB alone groups (11.2 ± 6.2 vs. 11.4 ± 5.8 months, *P* = 0.881). In the SB with PR group, retinal detachment involving ≥ three quadrants was a significant risk factor for surgical failure (hazard ratio, 3.04; *P* = 0.041). Adjuvant pneumatic retinopexy does not provide additional benefit in improving the surgical outcomes of SB for primary RRD repair.

## Introduction

Rhegmatogenous retinal detachment (RRD) occurs when a break or hole in the retina allows fluid to accumulate in the subretinal space, resulting in separation of the neurosensory retina from the underlying retinal pigment epithelium (RPE). RRD is an important cause of visual loss, with an annual incidence ranging from 6.3 to 18.9 per 100,000 individuals^1–5^.

Scleral buckling (SB), a surgical technique used for RRD repair, relieves vitreous traction on the retinal break by bringing the RPE and retina close to each other, which subsequently leads to occlusion of the retinal break^6–8^. Primary SB success rates of over 80% have been reported in previous studies^9–11^.

Pneumatic retinopexy (PR), first described by Hilton and Grizzard^12^, is a fast, convenient, and minimally invasive technique used for the management of RRD. In PR, a gas bubble is used to occlude the retinal break by utilizing its expanding and buoyant properties, thereby impeding the flow of intraocular fluid into the subretinal space^13,14^. However, PR alone is not widely used in clinical practice because of its limited treatment criteria, which include single or clustered multiple breaks within one clock hour in the superior 2/3 of the fundus (from 8 to 4 o'clock), and its relatively low success rate^15,16^.

When SB and PR are performed simultaneously for the treatment of primary RRD, better surgical outcomes may be expected owing to the additive effect of each therapeutic option. However, there is currently no study on systematic comparison of the surgical outcomes of SB with PR and SB alone. Therefore, the aim of this study was to compare the efficacy of SB with adjuvant PR versus SB alone for the treatment of primary RRD, and to investigate the risk factors for surgical failure after SB and SB with PR.

## Results

A total of 249 patients were included in the study: Of these, 88 underwent SB with PR, whereas 161 underwent SB alone. The mean follow-up duration was 23.1 ± 13.8 months. The SB with PR group had a significantly older mean age (49.0 ± 13.1 years vs. 40.2 ± 16.1 years, *P <* 0.001), shorter time to symptom onset (5.3 ± 5.9 days vs. 8.8 ± 11.0 days, *P =* 0.009), and higher proportion of patients with superior retinal tears (96.6% vs. 51.6%, *P <* 0.001) than the SB alone group. The demographic and clinical characteristics of each group are shown in Table 1. The mean best-corrected visual acuity (BCVA) significantly improved after surgery in both the SB with PR group and the SB alone group (P < 0.001, respectively; Table 2). Additionally, there was no significant difference in postoperative BCVA between the SB with PR group and the SB alone group (*P =* 0.661).

**Table 1:** Demographic data and intraoperative variables of patients who underwent scleral buckling with adjuvant pneumatic retinopexy (SB with PR) and scleral buckling (SB) alone.

Variable	Total (n = 249)	SB with PR (n = 88)	SB alone (n = 161)	*P* value
Age, years	43.3 ± 15.7	49.0 ± 13.1	40.2 ± 16.1	< 0.001*
Sex				0.217
Male	132 (53.0)	42 (47.7)	90 (55.9)	
Female	117 (47.0)	46 (52.3)	71 (44.1)	
Axial length, mm	25.69 ± 1.82	25.60 ± 1.92	25.72 ± 1.78	0.682
Lens status				0.203
Phakic	238 (95.6)	82 (93.2)	156 (96.9)	
Pseudophakic	11 (4.4)	6 (6.8)	5 (3.1)	
Spherical equivalent, D	−3.45 ± 3.77	−3.48 ± 4.28	−3.44 ± 3.52	0.948
Preoperative IOP, mmHg	11.6 ± 3.7	11.8 ± 3.5	11.4 ± 3.9	0.506
Right eye	133 (53.4)	51 (58.0)	82 (50.9)	0.288
Onset, days	7.4 ± 9.5	5.3 ± 5.9	8.8 ± 11.0	0.009*
Follow-up, months	23.1 ± 13.8	24.4 ± 13.4	22.3 ± 14.0	0.259
Operation time, minutes	107.1 ± 44.8	103.2 ± 43.2	109.3 ± 45.6	0.310
Macula status				0.189
Macula-on	136 (54.6)	53 (60.2)	83 (51.6)	
Macula-off	113 (45.4)	35 (39.8)	78 (48.5)	
Quadrant of RD				0.136
1	40 (16.1)	16 (18.2)	24 (14.9)	
2	158 (63.5)	56 (63.6)	102 (63.4)	
3	39 (15.7)	16 (18.2)	23 (14.3)	
4	12 (4.8)	0 (0.0)	12 (7.5)	
Number of tears				0.134
Single	166 (66.7)	64 (72.7)	102 (63.4)	
Multiple	83 (33.3)	24 (27.3)	59 (36.6)	
Location of tear(s)				<0.001*
Superior	168 (67.5)	85 (96.6)	83 (51.6)	
Inferior	68 (27.3)	3 (3.4)	65 (40.4)	
Combined	13 (5.2)	0 (0.0)	13 (8.1)	
PVR grade				0.407
No PVR	203 (81.5)	76 (86.4)	128 (79.5)	
Grade A	10 (4.0)	0 (0)	9 (5.6)	
Grade B	36 (14.5)	12 (13.6)	24 (14.9)	
Gas tamponade				
Air	37 (42.1)	37 (42.1)	–	
SF_6_	2 (2.3)	2 (2.3)	–	
C_3_F_8_	49 (55.7)	49 (55.7)	–	

Data are expressed as mean ± SD or number (%) of eyes.

*BCVA* best-corrected visual acuity, *D* diopters, *IOP* intraocular pressure, *LogMAR* logarithm of the minimal angle of resolution, *PVR* proliferative vitreoretinopathy, *RD* retinal detachment.

^*^P value < 0.05.

**Table 2:** Best-corrected visual acuity (BCVA) before and after surgery according to the type of surgery.

Variable	Total (n = 249)	SB with PR (n = 88)	SB alone (n = 161)	*P* value
Preoperative BCVA, logMAR	0.67 ± 0.82	0.74 ± 0.92	0.64 ± 0.75	0.350
Postoperative BCVA, logMAR	0.19 ± 0.35	0.39 ± 0.04	0.33 ± 0.03	0.661
*P* value^†^	<0.001	<0.001	<0.001	

*BCVA* best-corrected visual acuity, *LogMAR* logarithm of the minimal angle of resolution, *PR* pneumatic retinopexy, *SB* scleral buckling.

^†^Paired t test.

**Figure 1:**
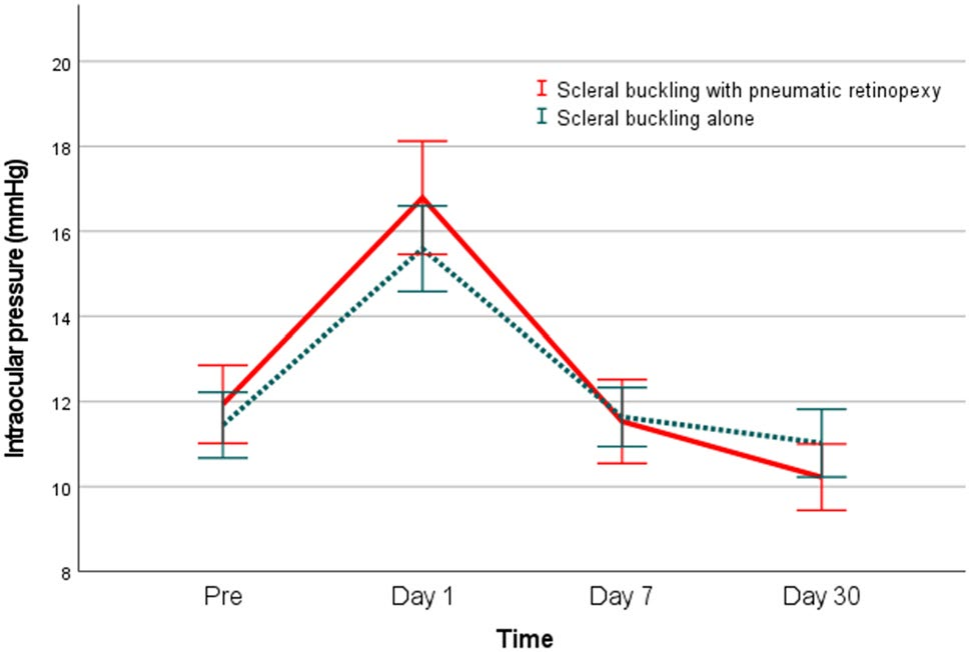
Preoperative and postoperative changes in intraocular pressure.

There was no significant difference in primary anatomical success rate between the SB with PR and SB alone groups (72 of 88 [81.8%] vs. 130 of 161 [80.7%], *P* = 0.836; Table 3). In addition, there was no significant difference in final anatomical success rate between the two groups (87 of 88 [98.9%] vs. 161 of 161 [100.0%], *P* = 0.353). One patient in the SB with PR group refused further treatment, which ultimately resulted in failed retinal reattachment. The proportion of patients with new tears as the cause of primary anatomical failure was significantly higher in the SB with PR group than in the SB alone group (10 of 16 [62.5%] vs. 4 of 31 [12.9%], *P* = 0.001). The proportion of patients with an inadequate buckle was significantly lower in the SB with PR group than in the SB alone group (2 of 16 [12.5%] vs. 22 of 31 [71.0%], *P* < 0.001). Among the patients who achieved primary anatomical success, the number of those who showed postoperative epiretinal membrane (ERM) formation was significantly higher in the SB with PR group than in the SB alone group (11 of 72 [15.3%] vs. 6 of 130 [4.6%], *P* = 0.009). None of the patients who had postoperative ERM required further surgical intervention. Patients in both groups showed elevated intraocular pressure (IOP) on postoperative day 1; however, there was no significant difference between the two groups (4.9 mmHg vs. 4.1 mmHg, *P* = 0.478). Subsequently, the elevated IOP gradually decreased. The proportion of patients with IOP ≥ 21 mmHg during the 30-day postoperative period was higher in the SB with PR group than in the SB alone group; however, the difference between the two groups was not significant (12 of 72 [16.7%] vs. 11 of 130 [8.5%], *P* = 0.079). In all 23 cases, IOP was normalized after the instillation of IOP-lowering agents. There was no significant difference in mean preoperative and postoperative IOP between the two groups at any timepoint (Fig. 1).

**Table 3:** Surgical outcomes and postoperative complications of scleral buckling with adjuvant pneumatic retinopexy (SB with PR) and scleral buckling (SB) alone.

Variable	Total (n = 249)	SB with PR (n = 88)	SB alone (n = 161)	*P* value
Primary anatomical success	202 (81.1)	72 (81.8)	130 (80.7)	0.836
Final anatomical success	248 (99.6)	87 (98.9)	161 (100)	0.353
Cause of primary failure				
New break	21 (44.7)	10 (62.5)	4 (12.9)	0.001*
Inadequate buckle	17 (36.2)	2 (12.5)	22 (71.0)	<0.001
Proliferative vitreoretinopathy	8 (17.0)	3 (18.8)	5 (16.1)	1.000
Macular hole	1 (2.1)	1 (6.3)	0 (0.0)	0.340
Complications^†^				
Increased IOP (≥21mmHg)	23 (11.4)	12 (16.7)	11 (8.5)	0.079
Epiretinal membrane	17 (8.4)	11 (15.3)	6 (4.6)	0.009*
Macular hole	1 (0.5)	1 (1.4)	0 (0.0)	0.356
Cystic macular edema	1 (0.5)	0 (0.0)	1 (0.8)	1.000
Cataract^‡^	0 (0.0)	0 (0.0)	0 (0.0)	

Data are expressed as the number (%) of eyes.

*IOP* intraocular pressure.

^†^Among patients who achieved primary anatomical success.

^‡^Patients who underwent cataract surgery within 6 months after primary surgery.

*P value < 0.05.

In the entire study cohort, preoperative macula-off retinal detachment (RD) was a significant risk factor for primary anatomical failure (hazard ratio [HR], 1.87; *P* = 0.036), whereas gas tamponade was not (HR, 0.91; *P* = 0.748) (Table 4). The univariate Cox regression analysis showed that in the SB with PR group, RD involving ≥ three quadrants (HR, 4.51; *P* = 0.003) and preoperative macula-off status (HR, 3.80; *P* = 0.013) were significant risk factors for primary anatomical failure. However, the multivariate Cox regression analysis showed that only RD involving ≥ three quadrants was a significant risk factor primary anatomical failure (HR, 3.04; *P* = 0.041; Table 5). The subgroup analysis revealed that the use of expandable gases (C_3_F_8_ and SF_6_) was not associated with primary anatomical failure (HR, 2.41; *P* = 0.127).

**Table 4:** Cox proportional hazard analysis of factors associated with primary anatomical failure of scleral buckling with adjuvant pneumatic retinopexy and scleral buckling alone.

Variable	Univariate		Multivariate	
	HR (95% CI)	*P* value	HR (95% CI)	*P* value
Demographics				
Age	1.01 (0.99–1.03)	0.209		
Female	0.60 (0.33–1.09)	0.093	0.64 (0.35–1.16)	0.142
Onset	0.97 (0.92–1.01)	0.140		
Ocular characteristics				
Quadrant				
Quadrant 1, 2	Reference (1)			
Quadrant 3, 4	1.76 (0.94–3.28)	0.078	1.49 (0.78–2.86)	0.213
Macula off RD	1.85 (1.03–3.31)	0.039*	1.87 (1.04–3.34)	0.036*
PVR grade				
≤ Grade A	Reference (1)			
Grade B	0.67 (0.27–1.69)	0.396		
Number of tears				
Single	Reference (1)			
Multiple	1.16 (0.64–2.11)	0.618		
Location of tear(s)				
Superior	Reference (1)			
Inferior	1.04 (0.54–1.97)	0.918		
Combined	0.80 (0.19–3.33)	0.757		
Gas tamponade	0.91 (0.50–1.66)	0.748		

*CI* confidence interval, *HR* hazard ratio, *PVR* proliferative vitreoretinopathy, *RD* retinal detachment.

^*^P value < 0.05.

**Table 5:** Cox proportional hazard analysis of factors associated with primary anatomical failure of scleral buckling with adjuvant pneumatic retinopathy.

Variable	Univariate		Multivariate	
	HR (95% CI)	*P* value	HR (95% CI)	*P* value
Demographics				
Age	0.99 (0.96–1.03)	0.765		
Female	0.51 (0.19–1.41)	0.194		
Onset	1.02 (0.94–1.10)	0.662		
Ocular characteristics				
Quadrant				
Quadrant 1, 2	Reference (1)			
Quadrant 3, 4	4.51 (1.67–12.16)	0.003*	3.04 (1.05–8.84)	0.041*
Macula off RD	3.80 (1.32–10.94)	0.013*	2.66 (0.85–8.32)	0.092
PVR grade				
≤ Grade A	Reference (1)			
Grade B	1.58 (0.45–5.56)	0.473		
Number of tears				
Single	Reference (1)			
Multiple	0.61 (0.14–2.68)	0.513		
Location of tear(s)				
Superior	Reference (1)			
Inferior	2.32 (0.31–17.61)	0.415		
Gas type				
Air	Reference (1)			
Expandable gas†	2.41 (0.78–7.48)	0.127		

*CI* confidence interval, *HR* hazard ratio, *PVR* proliferative vitreoretinopathy, *RD* retinal detachment.

^†^SF_6_ (n = 2) and C_3_F_8_ (n = 49).

^*^P value < 0.05.

The mean time to subretinal fluid (SRF) absorption was not significantly different between the SB with PR (n = 24) and SB alone (n = 55) groups (11.2 ± 6.2 months vs. 11.4 ± 5.8 months, *P* = 0.881).

In SB with PR, the primary anatomical success rates varied according to the gas type: Air (33 of 37 [89.2%]), SF_6_ (1 of 2 [50.0%]), C_3_F_8_ (38 of 49 [77.6%]), with no significant differences observed between the groups (Air vs. SF_6_, *P* = 0.243; Air vs. C_3_F_8_, *P* = 0.159; SF_6_ vs. C_3_F_8_, *P* = 0.419). The rates of ERM formation were as follows: Air (2 of 35 [5.4%]), SF_6_ (0 of 2 [0.0%]), C_3_F_8_ (9 of 40 [18.4%]), with no significant differences observed between the groups (Air vs. SF_6_, *P* = 1.000; Air vs. C_3_F_8_, *P* = 0.105; SF_6_ vs. C_3_F_8_, *P* = 1.000).

## Discussion

In this study, we investigated the surgical outcomes of SB with adjuvant PR for the treatment of primary RD. The results showed that the combination of SB and PR did not significantly improve reattachment rate compared to SB alone. In addition, SB with PR was associated with an increased risk of postoperative ERM formation. Furthermore, the time to SRF absorption was not significantly different between the SB with PR and SB alone groups. The results also showed that macula-off RD and RD involving ≥ three quadrants had a significantly negative impact on reattachment rate after a single surgery.

The efficacy of SB with PR for primary RD has been reported in previous studies. The authors of some studies introduced a technique called "D-ACE,” which involves drainage, air injection, cryotherapy, and explant surgery sequence, and reported primary anatomical success rates ranging from 85% to 96% for the treatment of simple RD^17–19^. Cheng et al.^20^ performed short-term external buckling with PR for 31 patients with inferior retinal detachment and reported a 6-month primary anatomical success rate of 87.9%. However, the inclusion and exclusion criteria of these studies varied. In addition, a comparative analysis was not performed in any of the studies. We performed a comparative analysis of SB with PR versus SB alone. The results of the present indicated that the primary anatomical success rate in the SB with PR group was 81.8%, which was not significantly different from the 80.7% in the SB alone group.

Interestingly, the percentage of patients with an inadequate buckle as a cause of primary anatomical failure was significantly lower in the SB with PR group than in the SB alone group. One possible explanation for this is that the gas tamponade may have supplemented inadequate buckles in the eyes of patients in the SB with PR group. The percentage of patients with new tears was significantly higher in the SB with PR group than in the SB alone group. The development of new breaks may be attributed to vitreous traction complicated by excessive movement of the gas bubble in the vitreous cavity. Considering these findings, it appears that while PR may have the potential to compensate for an inadequate buckle effect, it may also have the adverse effect of causing new tears. Therefore, adjuvant PR does not appear to provide additional benefits for improving surgical outcomes.

Among the patients with primary anatomical success, the number of those who showed postoperative ERM formation was significantly higher in the SB with PR group than in the SB alone group. Decrease in the concentration of hyaluronic acid in the vitreous, tearing and distortion of the cortical vitreous lamellae, and breakdown of the blood-ocular barrier are known mechanisms for postoperative ERM formation^21,22^. Fabian et al.^23^ reported that the incidence of ERM among patients with RRD who underwent a single PR was 11.3%; however, it had no significant effect on final visual acuity. Similarly, 11 eyes (15.3%) in the present study developed ERM that did not require surgical intervention during the mean follow-up period of 29.4 months.

Among patients who achieved primary anatomical success after a single SB with PR, 12 showed an elevated IOP higher than 21 mmHg. Of these 12 patients, six received air, five received C_3_F_8_, and one received SF_6_ as the tamponade agent. In other studies, the incidence of elevated IOP after SB with PR ranged from 6.1% to 8.2%^13,20^, which is lower than the 16.7% recorded in the present study. However, the definition of elevated IOP in previous studies was based on a threshold of 25 or 30 mmHg. This contributed to the higher incidence of elevated IOP in the present study than in previous studies. Additionally, in all 12 patients in the present study, IOP was normalized after the instillation of IOP-lowering agents and no other complications were observed during the follow-up period. There was no significant difference in mean preoperative and postoperative IOP between the two groups at any timepoint (Fig. 1).

We expected that PR would facilitate early occlusion of retinal breaks and rapid absorption of SRF owing to its expanding and buoyant properties. However, the time to SRF absorption did not differ significantly between the SB with PR and SB alone groups. The delayed absorption of SRF may have been influenced by impairment of the RPE pump, as well as the protein-rich and highly viscous nature of the SRF. Several studies have indicated that it takes 10-26 months after PR for SRF to absorb^24–26^. In the present study, the mean time to complete absorption of SRF in the SB with PR group was 11.2 months, whereas that in the SB alone group was 11.4 months. These results are consistent with the findings of previous studies.

We analyzed the factors associated with primary anatomical failure in the entire cohort. We found that macula-off RD was a significant risk factor for primary anatomical failure. Several studies have also indicated that preoperative macula-off status is a significant risk factor for primary anatomical failure after SB^27–29^.

The multivariate Cox regression analysis showed that in the SB with PR group, RD involving ≥ three quadrants was a significant risk factor for primary anatomical failure. To the best of our knowledge, there have been no previous reports on the risk factors for primary anatomical failure in patients who underwent SB with PR. Similar to our results, a study on the efficacy of PR indicated RD involving 4.5 clock hours or more is a significant risk factor for anatomical failure, whereas macula status is not^30^.

Several studies have reported satisfactory results using filtered air injection during PR and have documented its effectiveness^31–33^. Specifically, in a double-blind, randomized, clinically controlled noninferiority trial, air injection showed no significant difference in success rates compared to C_3_F_8_, demonstrating noninferiority^34^. Therefore, we used filtered air or expandable gases for PR. In the present study, there was no significant difference in the primary anatomical success rates based on gas type, in accordance with the results of previous studies^13,28^.

This study has several limitations. First, the retrospective design of the study and enrollment of participants from a single center may have introduced a selection bias. Second, because the decision to perform adjuvant gas tamponade was made at the discretion of the vitreoretinal fellow, there is a small possibility that an additional gas injection was administered to the worse eye, which may have influenced the surgical failure rates. However, the surgeons in this study frequently administered adjuvant gas injections in patients with superior RD, rather than depend on other ocular conditions, which could minimize selection bias. Additionally, SB surgeries for patients in the control group were performed by the same surgeons to increase the reliability of the comparisons between the two groups.

In conclusion, this study demonstrated that the combination of SB and PR does not improve anatomical success rate compared to SB alone. In addition, SB with PR is associated with an increased risk of postoperative ERM formation. Furthermore, the time to SRF absorption did not differ significantly between patients who underwent SB with PR and those who underwent SB alone.

## Methods

This retrospective observational study was conducted at Seoul National University Bundang Hospital. The study was conducted in accordance with the tenets of the Declaration of Helsinki. The study protocol was reviewed and approved by the Institutional Review Board of Seoul National University Bundang Hospital (No. B-2305-827-102). Due to the retrospective nature of the study, the requirement for informed consent was waived by the Institutional Review Board of Seoul National University Bundang Hospital.

### Patient eligibility

We reviewed the medical records of patients who underwent either SB with PR or SB alone for the treatment of primary RRD at Seoul National University Bundang Hospital between January 1, 2017, and February 28, 2021. Patients who completed at least 6 months of follow-up after the primary surgery were included. The patients were classified into the SB with PR group (n = 88) and the SB alone group (n = 161). The surgeries were performed by seven vitreoretinal fellows at our institution who had experience in performing more than 30 SB procedures. The decision regarding whether to perform adjuvant gas injection and the type of gas to be used was made at the discretion of the surgeon, with the expectation of additional benefits in terms of anatomical success.

The exclusion criteria were as follows: eyes with (1) RD caused by other factors, such as traction or exudation; (2) proliferative vitreoretinopathy (PVR) ≥ grade C; (3) history of ocular surgery, including vitrectomy and SB; or (4) history of ocular trauma. PVR was classified according to the Retina Society Classification guidelines^35^.

### Examination

The patients underwent a comprehensive ophthalmic examination the included the following: measurement of BCVA, measurement of IOP, slit-lamp biomicroscopy, fundus examination, widefield color fundus photography (Optos PLC, Dunfermline, UK), and spectral-domain optical coherence tomography (SD-OCT; Heidelberg Engineering, Heidelberg, Germany). Regular preoperative and postoperative follow-up, which included measurement of BCVA, wide fundus photography, and SD-OCT, were performed.

### Surgical procedures

**Figure 2:**
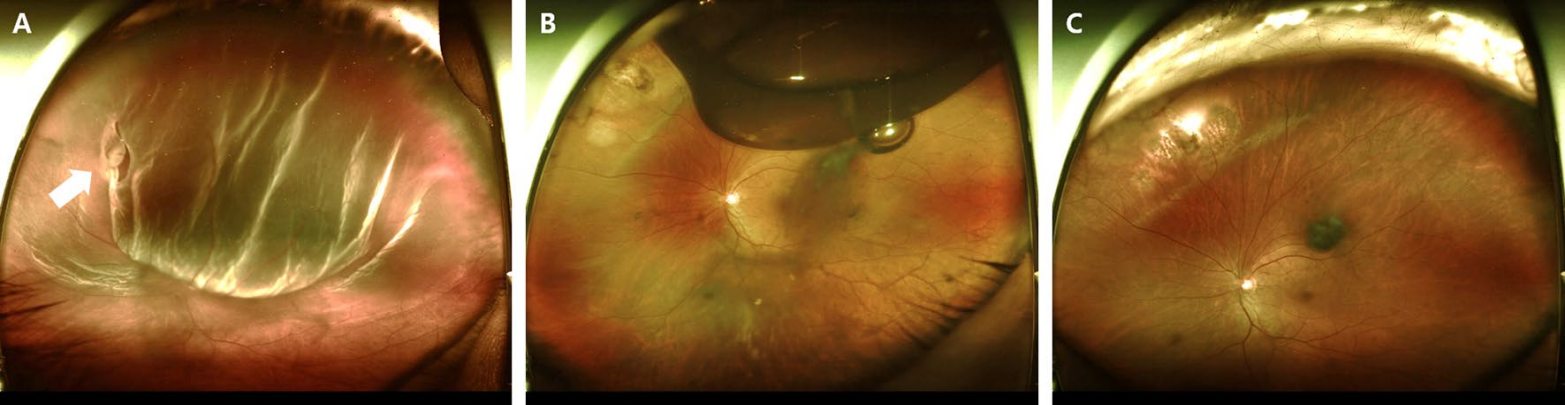
Wide fundus photography of primary rhegmatogenous retinal detachment before and after scleral buckling (SB) with pneumatic retinopexy (PR). (**A**) A 55-year-old man with superior bullous retinal detachment and a single retinal break at the 10 o’clock position (arrow). (**B**) Subretinal fluid was significantly reduced 1 week after SB with PR (C_3_F_8_, 0.3cc). (**C**) The retina was reattached three months after surgery.

All surgeries were performed under general anesthesia and standard sterilization. Conjunctival peritomy was performed, followed by isolation of the rectus muscles using a 4-0 silk suture. Cryoretinopexy of the retinal breaks was performed using a cryoprobe. The explants were sutured onto the sclera using 5-0 ethibond mattress sutures. A silicone sponge (#506; MIRA Inc., Waltham, MA, USA) was placed for segmental buckling, and a silicone tire (#287; MIRA Inc., Waltham, MA, USA) with a silicone sleeve (#270; MIRA Inc., Waltham, MA, USA) was placed for 360° circumferential buckling. External drainage of SRF was performed in no case. For patients in the SB with PR group, ≥ 0.5 mL of filtered sterile air, ≥ 0.5 mL of 100% pure SF_6_, or ≥ 0.3 mL of 100% pure C_3_F_8_ was injected into the vitreous cavity following SB. The gas bubble was injected at the point 3.5 mm posterior to the limbus using a syringe with a 30-gauge needle (Figure 2). If IOP notably increased after the gas injection, anterior chamber paracentesis (generally ≥ 0.3 mL) was performed as needed. The patients in the SB with PR group were positioned appropriately for at least 1 week so that the gas adequately covered the retinal tear.

### Outcome measures

We defined primary anatomical success as retinal reattachment at 6 months after a single surgery, and final anatomical success as retinal reattachment at the end of follow-up. The SD-OCT scan parameters used are as follows: pattern size 30° × 20° (9.1 × 6.0 mm) and 25 sections (251 μm between B-scans). SRF was defined as separation of the neurosensory retina from the RPE by fluid detected using SD-OCT volumes. We defined SRF absorption as the absence of fluid on the SD-OCT images. Postoperative complications were analyzed only in patients who achieved primary anatomical success because the reoperation itself may have affected the postoperative outcomes. We performed a comparative analysis of the mean time to SRF absorption in patients with macula-off RD who achieved primary anatomical success.

### Statistical analysis

Statistical analyses were performed using SPSS version 27.0 software (SPSS Inc, Chicago, Illinois, USA). We used the chi-square test and independent t-test to compare variables between the SB with PR and SB alone groups. In addition, we performed univariate Cox regression analysis to identify the factors associated with primary anatomical failure. Thereafter, we performed a multivariate Cox regression analysis of factors that showed statistical significance (*P* < 0.1) in the univariate analysis. Statistical significance was defined as a *P* value less than 0.05.

## Data Availability

All data generated or analyzed during this study are included in this published article.
